# The Management of the Pediatric Neurogenic Bladder

**DOI:** 10.1007/s11884-016-0371-6

**Published:** 2016-07-02

**Authors:** Renea M. Sturm, Earl Y. Cheng

**Affiliations:** 1 Division of Urology, Ann and Robert H. Lurie Children’s Hospital of Chicago, 225 E. Chicago Ave, Box 24, Chicago, IL 60611 USA; 2 Department of Urology, Feinberg School of Medicine at Northwestern University, 303 E. Chicago Ave, Chicago, IL 60611 USA

**Keywords:** Urinary bladder neurogenic, Pediatrics, Urology, Urinary bladder, Urinary incontinence

## Abstract

Neurogenic bladder is a heterogeneous entity that may result from a variety of conditions affecting the central or peripheral nervous systems. Regardless of etiology, the overall goals of management are primarily twofold. As a neurogenic bladder may affect the ability to store urine safely and to empty the bladder efficiently, early management is focused on optimization of bladder storage function to prevent irreversible injury to either the upper or lower urinary tracts. In older children, this goal is added to the challenge of maximizing quality of life through achievement of urinary continence and independence in bladder management that continues into the transition to adulthood.

In this review, we seek to bring the reader up-to-date regarding management of the pediatric neurogenic bladder with a focus on literature published in the past year. We discuss key contributions related to fetal intervention for myelomeningocele, monitoring and medical management of the neurogenic bladder and prediction of postoperative outcomes. Put together, these studies highlight the continued need for further research to improve evidence-based medical and surgical decision-making strategies for children affected by neurogenic bladder.

## Introduction

The normal lower urinary tract allows for both coordinated low-pressure filling and periodic voluntary bladder emptying. Given the complexity of its neural control, central or peripheral nervous system lesions that affect the parasympathetic, sympathetic or somatic systems may have varied effects on the lower urinary tract. Thus, a neurogenic bladder in children may occur secondary to central nervous system (CNS) lesions such as cerebral palsy, spinal dysraphisms and spinal cord injury or secondary to pelvic pathology such as tumors and anorectal malformations.

The goals in management of the neurogenic bladder are primarily twofold. As a neurogenic bladder may affect the ability to store urine safely and to empty the bladder efficiently, early management is focused on optimization of bladder function to prevent irreversible morphological and functional injury to either the upper or lower urinary tracts. In older children, this goal is added to the challenge of maximizing quality of life through achievement of urinary continence and independence in bladder management.

In this review, we seek to bring the reader up-to-date regarding management of the pediatric neurogenic bladder with a focus on literature published in the past year. We will discuss key contributions related to fetal intervention for myelomeningocele, monitoring and medical management of the neurogenic bladder and prediction of postoperative outcomes. Put together, these studies highlight the continued need for further research to improve evidence-based medical and surgical decision-making strategies for children affected by neurogenic bladder.

## Myelomeningocele: Fetal Surgery and Urinary Tract Outcomes

Although an initial decline in incidence of myelodysplasia was observed following widespread folate supplementation, the incidence of 3.7 per 10,000 live births in the United States [1] has stabilized during the past decade [2]. Given its substantial long-term morbidity and potential to impact neurologic development, prenatal closure of the open myelomeningocele defect has been initiated in specialized centers. While fetal closure has been associated with improvement in hydrocephalus and motor function, early reports of its effect on the urinary tract have been less promising [3–5].

The Management of Myelomeningocele Study (MOMS) was a randomized prospective clinical trial evaluating prenatal versus neonatal closure of open myelomeningocele. Early trial outcomes included decreased CSF shunt placement rates and improved mental development and motor function measures at age 30 months following in utero closure [6]. However, a subsequent evaluation of urologic outcomes found no difference in the rates of children meeting criteria for initiation of clean intermittent catheterization (CIC) by 30 months (38 versus 51 % of the prenatal and postnatal surgery groups respectively; RR 0.74, 95 % CI 0.48–1.12). Secondary effects included less trabeculation in the prenatal surgery cohort and, after adjustment for gender and lesion level, a decrease in the presence of bladder diverticulae and open bladder neck during filling [7•].

In the past year, two additional institutional series evaluating urinary tract outcomes following prenatal myelomeningocele closure were published. In the first series, only two of 48 infants at a mean age of 5.4 months had a normal bladder on urodynamics while findings that may place the upper tracts at increased risk (end fill or detrusor leak point pressure (EFP or DLPP) of >40 cm/H_2_0) were present in 56 %. Furthermore, upper tract changes were present early with hydronephrosis or vesicoureteral reflux (VUR) observed in 26 % [8]. In a second series of 58 children followed up to 5 years after prenatal closure, only 19 % were successfully toilet-trained [9]. Additional long-term assessment will continue to provide information regarding the effects of in utero closure on the urinary tract. In the interim, these findings emphasize the importance of educating parents that ongoing urologic assessment and management are essential for children post prenatal myelomeningocele closure as they remain at significant risk for the development of a neurogenic bladder.

## Radiologic and Urodynamic Assessment of the Neurogenic Bladder

The current options available to evaluate the neurogenic bladder and upper tracts include ultrasonography (US), fluoroscopy, nuclear medicine studies or urodynamic testing. As was discussed in a recent review regarding the urologic management of spina bifida [10••], there is a current debate regarding the optimal role and timing of these studies fueled by a desire to prevent excessive invasive testing and radiation exposure. This debate is reflected in recent literature that has evaluated the diagnostic and prognostic value of each test including the use of nuclear medicine studies versus US in evaluation of the upper urinary tract, non-invasive methods of bladder wall assessment and urodynamics in children undergoing tethered cord release (TCR).

## Evaluation of Renal Scarring: DMSA Versus US

Regardless of management strategy, the goal to detect early upper tract changes that may precede deterioration is key to long-term prevention of renal failure. Prior series of children with myelodysplasia have demonstrated rates of renal scarring or functional loss of 10-32 % on DMSA nuclear medicine scans [11–14]. As a follow-up study, 122 adults with spina bifida were evaluated with both DMSA and US modalities to obtain a baseline assessment of renal scarring. A significant difference in renal scar detection was observed between US (10 %) and DMSA scans (46 %). Additionally, hypertension was only correlated with the presence of scarring on DMSA in this adult population [15•]. Recent literature evaluating the optimal imaging strategy following a febrile UTI in children likewise demonstrated that even the most optimized current model based on US findings has a low predictive value for either renal parenchymal anomalies or the presence of VUR [16]. Although the majority of these studies evaluated children with non-neurogenic bladders, [17] the lack of sensitivity of US for either VUR or renal scarring is of particular interest in neurogenic bladder given the need to prevent irreversible renal damage and the association of VUR with renal cortical loss in these children [11–14]. These findings require future evaluation but call into question the premise of an evaluation strategy that is reliant on US changes to precede further testing or intervention.

## Non-invasive Monitoring of Lower Urinary Tract Function

There is ongoing need for improved methodologies to assess early alterations in the bladder wall that may portend development of poor compliance. Bladder wall hypertrophy with its associated morphologic changes may decrease bladder compliance through effects on smooth muscle, connective tissue, innervation and tissue hypoxia. The ultrasonographic assessment of the bladder wall as a clinically meaningful outcome requires the ability to reliably assess these changes early when an intervention may reverse or halt progression toward irreversible decompensation [18]. In the largest recent study evaluating the utility of bladder wall measurements by US in the neurogenic bladder, 272 adult patients with SCI were evaluated. Although no threshold wall thickness was predictive of urodynamic findings, BWT was significantly increased in individuals with neurogenic detrusor overactivity associated with detrusor sphincter dyssynergia (NDO/DSD) or impaired bladder compliance compared to those without DSD [19]. On the other hand, in the actively managed pediatric neurogenic bladder, this predictive capacity of BWT may be more elusive as demonstrated by multiple series with mixed results [20–22]. In particular, a recent study of 53 closely managed children with spina bifida found that BWT was not predictive of unfavorable urodynamic parameters even when measured at specified bladder volumes [23•]. To improve the predictive capability of non-invasive studies of the lower urinary tract, our center is currently evaluating alternative technologies including bladder elastography as a potential solution to the ongoing need for a non-invasive evaluation strategy for these children.

## Predictive Urologic Changes in Tethered Cord Release

Urologic findings are often the first clinical sign in the up to one third of individuals who develop symptomatic tethering on long-term follow-up after postnatal myelomeningocele closure [24]. Two series were published in reference to secondary tethering and urologic outcomes of children in the past year. In a series of 23 children with secondary tethered cord, TCR significantly improved bladder compliance, DLPP and EFP. Urologic symptoms were improved in 62 % with all previously incontinent children reporting resolved or improved continence postoperatively [25]. These findings added to a growing body of literature evaluating clinical outcomes following primary TCR in occult spinal dysraphism. In a recent series from our institution, although asymptomatic children with cutaneous lesions had excellent continence rates post-TCR, no specific urodynamic parameters were associated with urologic outcomes [26]. Similarly, in a second series of 40 children although significant improvement in both continence and UTI rates were observed following secondary TCR, preoperative neurologic symptoms prompting TCR were not a predictor of urologic outcomes [27]. Taken together, an improvement in bladder dynamics or symptoms may be observed following either primary or secondary TCR. However, as the current ability to predict such changes is limited, urologic follow-up with a postoperative urodynamic evaluation may be warranted following these procedures.

## Management of the Neurogenic Bladder

Prior to the widespread utilization of CIC for the management of neurogenic bladder, renal failure and urosepsis were common causes of long-term morbidity and mortality. Recent baseline data of 2172 participants in the National Spina Bifida Registry provides a window into current management. In the specialized centers participating, 74 % of participants with myelomeningocele and bladder impairment utilized CIC [28]. Fortunately, in this era of CIC incorporation into practice, long-term success has been achieved in preventing VUR and hydronephrosis, decreasing augmentation cystoplasty (AC) rates and reducing postoperative mortality secondary to renal failure [29–31]. The challenge remains, however, to identify children who would benefit from initiation of CIC and the optimal timing for each medical or surgical intervention currently available.

## Medical Management

In a stepwise process of neurogenic bladder management, initial efforts at maintaining a compliant storage reservoir and treatment of urinary continence are focused on improved bladder drainage through catheterization and the addition of medications in an individualized manner. If the desired result is not achieved through anticholinergic therapy, another combination of medications, endoscopic management or neuromodulation may be utilized prior to surgical reconstructive procedures. Recent studies have focused on medical management alternatives and the efficacy of intravesical Botox for these children.

## Anticholinergics and Pharmacologic Therapy

Anticholinergic therapy has been demonstrated to have both short- and long-term effects on the bladder. A longitudinal study of 121 children with neurogenic bladder treated for a median duration of 19 months found that oral oxybutynin had sustained effects on both continence and bladder compliance. Of the 51 children with a pretreatment EFP of >40 cm/H_2_0, mean EFP decreased to and remained <40 cm/H_2_0 in 63 % throughout treatment. Oxybutynin was well tolerated with only six discontinuations due to AEs [32].

As an alternative route of administration, intravesical oxybutynin has also been evaluated for its potential to decrease the side effects of anticholinergic therapy by reducing first pass metabolism in the liver while maintaining high systemic efficacy and bioavailability [33–36]. A recent publication reported 15-year mean follow-up following transition from oral to intravesical oxybutynin for pediatric NGB in ten children with DSD. Ongoing suppression of detrusor overactivity, maintenance of long-term effects on bladder compliance (30 % at onset versus 90 % at last follow-up had an EFP <40 cm/H_2_0) and increased bladder capacity (from 5th to 25–50th percentile for age) was observed. Intravesical oxybutynin was well-tolerated; no side effects were reported [37••].

Finally, medication alternatives to anticholinergic therapy are being evaluated. A recent study highlighted the potential for further targeting of the adrenergic pathway by finding an association between children whose LUTS failed to respond to anticholinergics and genetic polymorphisms in the adrenergic rather than cholinergic pathway [38]. An example of an alternative agent that targets adrenergic transmission is mirabegron, a β3 agonist demonstrated to have efficacy in adults with NDO due to SCI with significant decreases in EFP, frequency of evacuation and incontinence episodes [39]. Although anticholinergic therapy is currently the mainstay of medical management for pediatric NGB, further studies may provide additional options for medical management, including the potential for targeted pharmacologic manipulation of the adrenergic pathway in children with NDO.

## Intravesical Botulinum Toxin A

Botulinum toxin A was FDA approved in adults for intravesical injection in the treatment of urinary incontinence due to NDO. Prospective assessments in children with NGB have likewise demonstrated significant improvement in continence, bladder capacity and compliance following Botox injection [40, 41]. In addition, a decrease in fibrosis after multiple injections has been described that may correspond to the potential for inhibition of detrimental remodeling in certain bladders [42].

Two retrospective series recently explored outcomes in the treatment of pediatric NGB and optimization of patient selection for intravesical Botox therapy. Khan et al. reviewed the experience at a single center of children with NDO who were either anticholinergic refractory or intolerant [43•]. Following injection of 300U into the detrusor, 54 % of children reported improved continence. A corresponding increase in mean cystometric capacity and a decrease in EFP and frequency of uninhibited detrusor contractions were observed. Mean duration of clinical effectiveness was 4.6 months (range 0–18). Of note, continence rates appeared to be highest in the subset of children with anticholinergic intolerance (75 %) versus those refractory to medical management (50 %) following Botox injection. Although interpretation remains inconclusive as the number within the cohort intolerant of anticholinergic therapy was small, it was hypothesized that these children may represent a subset less likely to have a fibrotic bladder [43•]. This observation aligns with publications by Tiryaki [44], Kim [45], and Kask et al. [46] which all found that severely impaired bladder compliance, particularly in the presence of a lack of detrusor activity, portends the poorest prognosis for response to intravesical botox injections in children with NGB. In these series, children with NDO and mild to moderate changes in compliance had a better response both in terms of continence and bladder dynamics following intravesical botox injection as compared to those with more severe compliance changes.

Regardless of the efficacy of a single intravesical Botox treatment for these children, however, a follow-up question recently addressed was its utility as a chronic management strategy. In 22 children with NDO who underwent a total of 62 intravesical Botox injections over a 10-year period, the median response time was 7 months (range 0–25) with a stable response observed up to a 7th injection [47••]. This aligns with a former series of eight children with NGB whose pre and postoperative urodynamics demonstrated a mean EFP decrease to <40 cm/H_2_0 and a consistent increase in bladder capacity after each of three repeated injections [48]. Although not currently FDA approved for treatment of NDO in children, Botox is a promising early alternative to augmentation, particularly for those individuals who are intolerant of anticholinergic therapy. Further studies are anticipated to continue to elucidate the optimal candidates, timing and utility of early treatment with Botox in children with NDO.

## Surgical Reconstruction

When medical and intravesical options fail to provide satisfactory results, surgical reconstruction may be required to maintain low intravesical storage pressure and achieve treatment goals for urinary continence. Current options for surgical management include incontinent diversion for those who are not candidates for CIC or individualized combinations of augmentation cystoplasty (AC), a bladder outlet procedure such as bladder neck reconstruction (BNR), sling, artificial urethral sphincter or bladder neck closure (BNC) and creation of a catheterizable channel [10••]. In the past year, literature contributions included analyses of enterocystoplasty (EC) outcomes, the selection of children for bladder neck reconstruction without concurrent bladder augmentation and the expanding role of robotics in reconstructive surgery.

## Outcomes Following Augmentation Cystoplasty

The most common tissue sources currently utilized for AC are autologous ileum or colon. Although typically effective in increasing bladder capacity and compliance, an EC is associated with both short- and long-term morbidity and requires lifelong urologic management. Two recent analyses of the American College of Surgeons’ National Surgical Quality Database (NSQIP) reported an approximately 30 % (23–33 %) 30-day overall event rate in children who underwent EC with or without appendicovesicostomy. The most common complications included urinary tract infections (9.6–10.7 %), wound complications (7.4–8.7 %), blood transfusions (6–6.1 %) and sepsis (2.8–3.5 %). Median length of stay was 8 days in both analyses with 30-day reoperative rates of up to 10 % and readmission rates of 13 % [49•, 50•]. In a separate study, when comorbidities were analyzed for contribution to perioperative outcomes in urologic bowel procedures, a BMI ≥95th percentile was found in 21 % of these children and was associated with both a fourfold increase in adjusted odds of overall wound complications [51] and an increased 30-day overall event rate [52].

In addition to short-term outcomes, 10-year cumulative complication incidence was calculated from the Pediatric Health Information System database in evaluation of 2831 augmentation cystoplasties. Causes of long-term morbidity included bladder rupture (2.9–6.4 %), small bowel obstruction (5.2–10.3 %), bladder stones (13.3–36 %) and pyelonephritis (16.1–37.1 %). Secondary surgery estimates included a 13.3–35.1 % rate of cystolitholapaxy and re-augmentation in 5.2–13.4 % [53]. In analysis of risk factors, an increased risk of bladder rupture and stone development was associated with bladder neck procedures and stoma creation at the time of augmentation [53].

Taken together, outcome studies such as these highlight both the importance of life-long urologic care following EC and the need for alternative management strategies. To date, bioengineered tissue alternatives have provided less than desirable human applicability with survival of large grafts limited in part by a lack of early vascularization leading to early functional loss and fibrosis [54, 55]. However, the significant morbidity associated with the use of autologous bowel for AC should continue to provide impetus for ongoing research to create viable tissue alternatives for these children.

## Outcomes of Bladder Neck Procedures Without Augmentation Cystoplasty

In the National Spina Bifida Patient Registry database, less than one third of children with spina bifida at their baseline registry evaluation were continent of either urine or stool [28]. For children with minimal outlet resistance who have not achieved the desired continence result via medical management, the surgical solution most often offered historically was a concurrent bladder neck reconstructive procedure, catheterizable channel and augmentation cystoplasty. In an attempt to address the morbidity of EC, multiple series have recently questioned the necessity of bladder augmentation in all cases [56].

In three recent series with 2 to 9 years of follow-up, children who underwent isolated bladder neck procedures proceeded to augment between 12 and 45 % of the time [57••, 58•, 59•]. Unfortunately, regardless of methodology utilized for urodynamic testing, no definitive preoperative urodynamic parameters were identified that could have predicted the response of each bladder to increased outlet resistance and the need for subsequent bladder neck or augmentation procedures. In the largest series, 18 % of 109 children who underwent a bladder outlet procedure without augmentation followed for a mean of 4.9 years had proceeded to AC. Although upper tract hydronephrosis or VUR resolved in all children in this series following AC, most concerning was the presence of new or increased renal scarring observed in 21 % of children evaluated both before and after an isolated bladder outlet procedure. Furthermore, the authors reported an estimated 10-year cumulative incidence of AC in 30 %, with over 50 % having developed upper tract changes and 20 % diagnosed with chronic kidney disease. Although significantly associated with the incidence of new or increased postoperative renal scarring, preoperative bladder compliance did not correlate with long-term success overall [57••]. Putting these series together, although there are bladders with myogenic failure and adequate compliance that may maintain low intravesical pressure following an isolated bladder neck procedure, the ability to preoperatively select optimal candidates for BNR without augmentation remains elusive.

## Robotic Approaches to Reconstructive Procedures

Surgical options continue to evolve with the application of a minimally invasive approach to an increasing number of reconstructive procedures. In the past year, several series have reported the feasibility of a robotic approach for AC, bladder neck reconstruction (BNR) and appendicovesicostomy. In a retrospective comparison of open (*n* = 13) versus robotic (15) ileocystoplasties with or without concomitant procedures, operative time was significantly longer (623 versus 287 min) while median length of stay was decreased (6 versus 8 days) in robotic as compared to open cohorts. Postoperative increase in bladder capacity, narcotic use and complication rates did not differ between the two approaches [60]. Similarly, feasibility was demonstrated of an entirely intracorporeal robotic EC with or without creation of catheterizable channels in a recent multi-institutional series of 22 adults [61]. Specific to the catheterizable channel, a single center experience of 39 robotic and 28 open appendicovesicostomies reported no difference in acute complication (26 and 29 %, respectively) or reoperation (29 versus 33 %) rates at a mean follow-up of 2.8 ± 2.5 years. However, the robotic series had higher rates of Clavien grade 2–3 complications including three early reoperations due to early bowel obstruction with internal hernia in the robotic cohort addressed by an altered technique with fixation of the channel and bladder to the abdominal wall [62]. Series such as these are needed to continue to define the long-term outcomes of robotic procedures with ongoing procedural optimization.

Robotic outcomes of bladder neck reconstruction have also recently been published. In one series, 38 robotic assisted Leadbetter-Mitchell type BNR and continent catheterizable channels without AC were performed. After a mean follow-up of 21 months, 82 % of individuals were dry with CIC which compares favorably with the 50–85 % continence rates published in the open literature [63]. In a second series, outcomes were compared between 19 robotic and 26 open bladder neck reconstructions. Operative time was significantly longer in the robotic cohort (8.2 versus 4.5 h) but no difference was observed in length of stay (median 4 days in each cohort) or acute complication rates (16 and 12 %, respectively). With a mean followup of 2.3 versus 3.2 years in the robotic versus open cohorts, there was no difference in rates of subsequent incontinence procedures performed in 42 and 56 % of children respectively [64].

As robotic procedures become increasingly common, recent studies have evaluated means to optimize perioperative care. A recent assessment of regional anesthesia options following robotic assisted procedures including transversus abdominal plane (TAP) and caudal blocks versus no regional block addressed these questions. A significant reduction in intraoperative opioid use and need for antiemetics was seen in children undergoing upper or lower tract urologic robotic surgery with a caudal block as compared to TAP or no regional anesthesia [65]. However, there was no significant difference in postoperative opioid use, maximum pain scores within 24 h postoperatively or length of hospital stay [65]. As surgical management options continue to evolve, ongoing optimization of surgical planning and perioperative management continues to be needed to refine techniques and outcomes for children with neurogenic bladder.

## Achieving Long-Term Goals: Quality of Life Assessment and Transition of Care

To evaluate in a systematic way whether the goals of individuals with neurogenic bladder are being met, several quality of life assessment tools have recently been evaluated including QUALAS-A [66] and QUALAS-C [67••] designed for adults and children with spina bifida as well as adolescent specific bowel [68] and bladder [69] functional measurement instruments. Although a complex relationship exists, qualitative interviews of youth and parents published in the past year reiterated the effect of continence on quality of life through greater independence and opportunities for social participation reported in children with minimal versus higher volume incontinence [70]. Development of assessment, educational tools [71] and options for bladder emptying [72] continue to refine our understanding to better address the needs of children with neurogenic bladder through facilitation of independence in bowel and bladder management.

Children with neurogenic bladder represent one of the largest pediatric populations in need of complex ongoing urologic management as they transition to adult care. Several studies that recently evaluated both youth with neurogenic baldder and their providers have emphasized the need for specialized treatment availability. In a survey of urologists, the majority (81 %) recommended that a urologist with training in adolescent/transitional care and experience in performance of genitourinary reconstructive procedures is ideally suited for the care of the mature patient with a complex genitourinary history [73]. Individuals who had completed this transition of urologic care were in agreement with the benefits of specialized management with 95 % considering their visit to a transitional care clinic beneficial [74]. Thoughtful planning in this process including assistance in navigation of an adult healthcare program and coordination with specialists who have expertise in this area will help to promote long-term genitourinary health for individuals with neurogenic bladder.

## Conclusion

The goals in management of the pediatric neurogenic bladder include preservation of the upper and lower urinary tracts and optimization of quality of life throughout childhood and in the transition to adulthood. As highlighted in this review of literature published in the past year, the techniques of assessment, medical and surgical management continue to evolve. Ongoing research to improve understanding of outcome determinants and optimal choice of candidates for each management strategy continues to be needed to minimize the significant current impact of the pediatric neurogenic bladder.
